# Albumin-corrected calcium ratios for citrate accumulation increase detection and improve mortality prediction in continuous kidney replacement therapy

**DOI:** 10.1186/s13054-026-05933-x

**Published:** 2026-03-11

**Authors:** Imran Khawaja, Naga Sumanth R. Gopireddy, Sahil Grover, Gwyndolyn Radford, Mony Fraer, Jonathan M. Nizar, Benjamin R. Griffin

**Affiliations:** 1 University of Iowa Hospitals and Clinics, Iowa City, IA USA; 2 Center for Access & Delivery Research and Evaluation (CADRE), Iowa City Veterans Affairs Health Care System, 200 Hawkins Drive, Iowa City, 52242 IA USA

Regional citrate anticoagulation (RCA) is widely used during continuous kidney replacement therapy (CKRT) because it prolongs circuit life and reduces bleeding risk compared to systemic anticoagulation [1]. Citrate accumulation (CA) (also known as citrate toxicity or citrate lock) is a feared complication occurring when the rate of citrate delivered systemically exceeds the body’s metabolic capacity resulting in systemic calcium chelation, often in the setting of severe liver dysfunction, shock, or multiorgan failure [2]. Because direct measurement of systemic citrate levels and underlying metabolic pathophysiology is not feasible in routine practice, CA is typically identified using the surrogate marker of total-to-ionized calcium ratio (tCa/iCa), with an empirically derived cutoff of > 2.5 used to identify clinically meaningful CA [3]. Most protocols calculate tCa/iCa using uncorrected total calcium [4]; however, low albumin levels will decrease total calcium but not ionized calcium, and because hypoalbuminemia is nearly universal among critically ill CKRT recipients [5], reliance on uncorrected total calcium may underestimate the presence of CA.

We conducted a retrospective analysis at the University of Iowa Hospitals and Clinics (IRB #202412279) examining 233 adults receiving RCA-CKRT between 2022 and 2024. By protocol, continuous venovenous hemodiafiltration was used for all patients with tCa/iCa measured 2 h after initiation and at least every 12 h thereafter. To minimize treatment-time and survivor bias, both uncorrected and albumin-corrected tCa/iCa ratios were calculated only from the first circuit. We compared CA rates defined using uncorrected tCa/iCa and albumin-corrected tCa/iCa, initially applying the conventional 2.5 threshold and subsequently determining optimized cutpoints using the Youden index. Associations with in-hospital mortality were evaluated using binary test metrics and multivariable logistic regression. Final variable models were determined using forward stepwise selection, with demographics, comorbidities, illness severity, and laboratory variables evaluated for inclusion.

Our cohort was critically ill with median sequential organ failure assessment (SOFA) scores of 12 (IQR 8–14) and substantial comorbidity including congestive heart failure (28%), diabetes (27%), chronic kidney disease (26%), and liver disease (17%). Shock etiology was often multifactorial; however, suspected sepsis appeared to be a predominant contributor with 73% of patients receiving at least one broad-spectrum antibiotic (vancomycin, cefepime, piperacillin-tazobactam, or a carbapenem). Patients were treated most often in the medical ICU (46%) followed by the surgical/neurosurgical ICU (38%) and cardiovascular ICU (16%). In-hospital mortality was 60%.

Hypoalbuminemia was highly prevalent, with a median albumin of 2.8 g/dL (IQR 2.4–3.2) and 205 (88%) patients with albumin values < 3.5 g/dL. Using uncorrected tCa/iCa values and a threshold of 2.5, only 12 patients (5%) met criteria for CA, with mortality in 10 (83%). Using an albumin-corrected tCa/iCa ratio identified 45 patients (19%), with mortality in 38 (84%). Albumin-corrected tCa/iCa correctly identified significantly more cases of mortality (27.1% vs. 7.1%, *p* < 0.001 by McNemar test). Among survivors (*n* = 93) specificity was modestly lower (93% vs. 98%, *p* = 0.06). Thus, albumin correction increased detection nearly four-fold while identifying a patient cohort with similarly high mortality risk.

Optimized thresholds for mortality prediction were 2.22 for the uncorrected ratio and 2.42 for the albumin-corrected ratio, and application of these cutpoints improved overall test performance (Table 1). In multivariable models, uncorrected tCa/iCa with a 2.5 threshold was not independently associated with mortality, whereas the other 3 approaches were. Collectively, these findings suggest that hypoalbuminemia may mask clinically significant CA, and that failure to account for albumin levels either through use of corrected tCa or a lower overall threshold may lead to systematic under recognition of high-risk patients.

These findings have clinical implications for RCA-CKRT management. Albumin correction, or alternatively adoption of a lower uncorrected cutoff, identifies a substantially larger group of patients with high mortality risk and may support closer laboratory monitoring and individualized CKRT adjustments in these patients. Whether CA directly contributes directly to mortality or simply serves as a marker of impaired metabolic reserve remains uncertain, as patients exceeding the corrected 2.5 ratio threshold exhibited higher illness severity, metabolic acidosis, thrombocytopenia, and liver dysfunction, consistent with prior reports [2]. However, low systemic ionized calcium is a known risk factor for arrhythmia, suggesting physiologic plausibility for clinical relevance. Prospective studies are needed to determine whether protocolized responses to these thresholds improve patient outcomes.

Our analysis has several limitations. The retrospective design does not account for clinical adjustments made after laboratory values were obtained. Analysis was restricted to first-circuit values to minimize treatment-time bias, but subsequent circuit management may have influenced outcomes. Practice patterns reflect a single center experience. Potentially useful laboratory values such as lactate are not routinely drawn as part of the standard CKRT protocol. Finally, the predominant Caucasian composition may limit generalizability to more racially diverse settings.

In conclusion, the traditional uncorrected tCa/iCa threshold of 2.5 may be overly conservative for identification of CA. Albumin correction of the tCa/iCa ratio or use of a lower uncorrected cutoff both substantially increase detection of CA and improve mortality risk stratification in patients receiving RCA-CKRT.

**Table 1:** Performance metrics of total-to-ionized calcium ratios for the prediction of in-hospital mortality in a cohort of 233 patients receiving regional citrate anticoagulation during continuous kidney replacement therapy

Test Metric	Uncorrected > 2.5 *N* = 12 (5.2%)	Uncorrected > 2.22 *N* = 53 (22.7%)	Corrected > 2.5 *N* = 45 (19.3%)	Corrected > 2.42 *N* = 66 (28.3%)
**Sensitivity (%)**	7 (3-11)	31 (24-39)	26 (19-33)	38 (30-46)
**Specificity (%)**	98 (95–100)	91 (85–97)	92 (86–98)	88 (81–94)
**PPV (%)**	83 (62–100)	85 (75–95)	84 (74–95)	83 (74–92)
**NPV (%)**	39 (33–45)	44 (37–52)	43 (36–50)	46 (39–54)
**Accuracy (%)**	41 (35–48)	54 (47–60)	51 (45–58)	57 (50–63)
**LR+**	3.0 (0.7–13.5)	3.4 (1.7–6.9)	3.3 (1.5–7.1)	3.0 (1.7–5.5)
**LR-**	0.95 (0.90-1.00)	0.76 (0.67–0.86)	0.80 (0.71–0.9)	0.71 (0.61–0.82)
**Univariable OR**	3.2 (0.7–14.9)	4.5 (2.0-10.1)	4.1 (1.8–9.7)	4.3 (2.1–8.8)
Multivariable OR*	3.9 (0.7–21.3)	5.3 (2.2–13.1)	3.4 (1.4–8.6)	4.5 (2.0-10.1)

PPV-positive predictive value; NPV-negative predictive value; LR+-positive likelihood ratio; LR- - negative likelihood ratio; OR-odds ratio

* Adjusted for sex, hemoglobin, and sequential organ failure assessment score

## Data Availability

The data that support the findings of this study are available from the corresponding author upon reasonable request.

## Abbreviations

CKRT: Continuous kidney replacement therapy
iCa: Ionized calcium
ICU: Intensive care unit
IQR: Interquartile range
IRB: Institutional review board
LR+: Positive likelihood ratio
LR–: Negative likelihood ratio
NPV: Negative predictive value
OR: Odds ratio
PPV: Positive predictive value
RCA: Regional citrate anticoagulation
SOFA: Sequential organ failure assessment
tCa/iCa: Total-to-ionized calcium ratio
